# School belonging and learning engagement among rural Chinese boarding high school students: the mediating role of academic buoyancy

**DOI:** 10.3389/fpsyg.2026.1958525

**Published:** 2026-10-19

**Authors:** Zhujun Zeng, Jian Huang, Yong Nie

**Affiliations:** Faculty of Education, Shaanxi Normal University, Xi’an, China

**Keywords:** academic buoyancy, learning engagement, mediation, rural boarding high school students, school belonging, self-determination theory

## Abstract

**Introduction:**

Rural boarding schools are common in China, but students in these schools often face academic difficulties due to limited family support. While school belonging predicts learning engagement, the mediating mechanisms remain unclear. Based on Self-Determination Theory, this study examines whether academic buoyancy mediates this relationship among rural boarding high school students.

**Methods:**

A cross-sectional survey was conducted with 657 students at a rural boarding high school in China. Validated scales measured school belonging, academic buoyancy, and learning engagement. Mediation analysis was performed using PROCESS Model 4, controlling for gender and grade.

**Results:**

School belonging, academic buoyancy, and learning engagement were at moderate levels and significantly positively correlated. Male students scored significantly higher in academic buoyancy than females, while school belonging was highest in Grade 1 and remained stable at a lower level in Grades 2 and 3, while learning engagement was higher in the senior grades than in Grade 1. Academic buoyancy partially mediated the relationship between school belonging and learning engagement.

**Discussion:**

These findings describe a statistical pathway from emotional connection, through the capacity to cope with routine academic setbacks, to behavioral engagement, offering empirical evidence for interventions to enhance rural boarding students’ learning engagement by strengthening school belonging and academic buoyancy.

## 1 Introduction

Rural boarding schools represent a vital educational model in China, offering access to education in remote regions. Yet this arrangement carries inherent challenges: prolonged separation from family, limited parental support, and heightened risks to mental health (Liu, 2012; Kleinfeld and Bloom, 1977; Mander et al., 2015) and academic adaptation (Li, 2016). In this context, the school transcends its traditional role, becoming students’ primary living community and source of social–emotional support.

Boarding provision is not unique to China, and its purpose varies across national settings. In several developed countries boarding operates primarily as a selective arrangement: German boarding schools are associated predominantly with elite education (Deppe, 2019), and in the United States preparatory programs are designed around entry to selective boarding institutions (Cox, 2018). Where boarding instead serves educational access in remote or under-resourced areas, the context is closer to the Chinese rural case. Indigenous Australian students from remote communities attend boarding schools because local secondary provision is unavailable; students report that boarding opens career pathways while also bringing homesickness and difficulties of self-concept and post-school transition (Macdonald et al., 2018). Australian comparisons of boarding with non-boarding students have reported higher anxiety and stress at the end of Grade 8 (Mander and Lester, 2017) and higher depressive symptoms among female boarders (Rudrum et al., 2022). Academic stressors are similarly prominent among boarding students in Malaysia (Wahab et al., 2013). The Chinese rural boarding context shares these features—prolonged family separation, concentrated academic pressure, and limited school resources—while adding the distinctive demands of the college entrance examination.

Boarding students perceive more support from teachers and less from parents than day-school peers, and peer-group integration may be harder to establish (Pfeiffer et al., 2016). Whether this shift undermines outcomes depends on how well the school compensates: where boarding and day students are taught under identical conditions their motivation, engagement and achievement are broadly at parity (Martin et al., 2021), and among boarding students specifically, perceptions of school safety and of the school environment are related to conduct problems, peer problems and inattention (Fredrick et al., 2022). A qualitative case study of graduates of a historically Black boarding school describes the school as instrumental in shaping their achievement, cultural esteem and sense of belonging (Alexander-Snow, 2011). In resource-constrained rural schools, however, how well the school provides this relational security cannot be assumed, making the quality of students’ connection to their school community a central concern.

For these students, school belonging—the sense of being accepted, respected, and valued within the school community (Goodenow, 1993), takes on profound significance. It is a fundamental human need that significantly influences individuals’ cognition, emotion, and behavior (Baumeister and Leary, 1995) and has been consistently linked to learning engagement (Gillen-O’Neel, 2021), the active investment of effort in academic pursuits. However, the psychological bridge between feeling connected and staying engaged remains underexplored—especially among boarding students navigating the dual pressures of academics and detachment from home.

Academic buoyancy, the ability to effectively cope with routine academic setbacks and challenges (Martin and Marsh, 2008), representing another important factor that may sustain high levels of engagement even in the face of failure (Collie et al., 2015), emerges as a promising candidate to explain this connection. Grounded in Self-Determination Theory (Ryan and Deci, 2017), we propose that belonging may provide the emotional security needed to foster such resilience, which in turn sustains consistent engagement over time. Yet this mediated pathway has not been examined in the distinct context of rural Chinese boarding schools, where belonging may hold unique cultural and emotional weight.

This study therefore investigates whether academic buoyancy mediates the relationship between school belonging and learning engagement among rural boarding high school students, seeking to illuminate the psychological process through which connection turns into commitment.

## 2 Theoretical framework

Self-Determination Theory (SDT) provides a comprehensive framework for understanding how school belonging may foster learning engagement through academic buoyancy. According to SDT, humans possess three innate psychological needs: autonomy, competence, and relatedness (Ryan and Deci, 2000). These needs are not only fundamental but also interdependent: their satisfaction constitutes a progressive and mutually reinforcing process (Ryan and Deci, 2017). Specifically, the fulfillment of one need, such as relatedness, can create conditions that support the satisfaction of the others (autonomy and competence).

In the context of rural boarding schools, where students experience limited family support, the school becomes a primary environment for satisfying these needs. Specifically, feelings of belonging satisfy the need for relatedness (Ryan and Deci, 2000), which provides individuals, particularly children, with a sense of safety. From this secure base, their exploratory tendencies can be expressed more robustly (Ryan and Deci, 2017),

This psychological security helps individuals overcome their anxiety and facilitates engagement (Ryan and Deci, 2017). The psychological resources liberated by relatedness satisfaction are precisely what fuel this buoyant capacity. In turn, students with high academic buoyancy—the capacity to effectively cope with routine academic setbacks and challenges (Martin and Marsh, 2008)—often engage in a set of strategies to mitigate academic stress (Putwain and Symes, 2016). They often possess more control over their academic trajectories(Collie et al., 2017), are less likely to experience learning burnout (Fu, 2024), are protected against the effects of minor academic adversities on achievement (Putwain et al., 2020), and thus demonstrate sustained learning engagement (Thomas and Allen, 2021).

In summary, grounded in SDT’s principles of basic psychological needs and their synergistic satisfaction, this study proposes that school belonging is associated with students’ academic buoyancy, thereby facilitating their sustained learning engagement. Specifically, school belonging satisfies the need for relatedness, establishing a secure relational base that frees psychological resources. These resources facilitate the adaptive appraisals and persistent coping strategies that define academic buoyancy—a construct embodying the activated states of competence and autonomy in the academic domain. This enhanced buoyancy conserves and channels motivational resources, ultimately promoting sustained behavioral engagement in learning.

## 3 Literature review and hypotheses

### 3.1 School belonging and learning engagement

School belonging refers to students’ perception of being accepted, respected, and supported within the school environment (Goodenow, 1993). As a fundamental human need, belonging significantly influences individuals’ cognition, emotion, and behavior (Baumeister and Leary, 1995), and has been consistently associated with students’ mental health and academic performance (Yuan et al., 2025). This need is particularly salient for children when they are away from their family environment (Kuang et al., 2019).

Learning engagement refers to students’ attention, commitment, and motivation toward the learning task (Jang, 2008). Empirical evidence has consistently demonstrated a significant positive correlation between school belonging and learning engagement (Yuan et al., 2025), with school belonging serving as a positive predictor of engagement (Gillen-O’Neel, 2021).

We posit that the relationship between school belonging and learning engagement may be particularly pronounced within China’s collectivist cultural context. Collectivism emphasizes group harmony, interdependence and relational obligations (Ryan and Deci, 2017). For rural boarding students who are physically separated from their primary family-based group, the school community often becomes their paramount in-group. Apart from being separated from their families and adapting to boarding life, students must also integrate into a closely-knit school community, each with its own unique group dynamics and operating mechanisms (Gottlieb, 2001). This cultural framework amplifies the motivational significance of belonging, making the school environment the primary source of both emotional sustenance and social identity for students facing limited family support. Consequently, school belonging assumes a crucial and multifaceted role in sustaining their learning motivation and engagement, even under adverse conditions.

Therefore, investigating the relationship between school belonging and learning engagement among high school students in rural Chinese boarding schools not only addresses a gap in the existing literature but also offers unique insights. This is particularly salient within the collectivist cultural context, where belonging is intertwined with group identity and social obligation, potentially amplifying its role as a key motivational resource within high-pressure, resource-limited educational environments.

### 3.2 Academic buoyancy as an intervening variable

Academic buoyancy, derived from positive psychology (PP), refers to students’ ability to successfully cope with typical academic setbacks and challenges in school life (Martin and Marsh, 2008). These typical academic difficulties encompass common academic challenges such as exam pressure, poor grades, difficult assignments, and tight deadlines (Martin and Marsh, 2008). According to the models of motivational resilience, environmental factors that fulfill learners’ basic psychological needs, including belonging, autonomy, and competence, contribute to greater emotional stability. This stability enables learners to remain calm and focused when facing difficulties (Pitzer and Skinner, 2017), thereby enhancing their ability to handle academic challenges.

The association between academic buoyancy and learning engagement holds across settings differing in language, curriculum and school system (Thomas and Allen, 2021; Ursin et al., 2021), and it appears in samples of teenagers in China, North America and the United Kingdom alike, with significantly stronger effects among the Chinese students (Martin et al., 2017). It is this stability across contexts, rather than any single estimate, that makes the same association reasonable to expect in a rural Chinese boarding population that none of these studies sampled. Furthermore, similar to many other positive psychology constructs, academic buoyancy is influenced by both internal factors (personality traits and variables) and external factors (situational issues) (Dong et al., 2022). According to SDT, students with a sense of school belonging tend to exhibit more resilient responses when facing setbacks and challenges (Reeve, 2012), indicating that a sense of belonging positively impacts academic buoyancy.

Based on SDT, this study proposes that school belonging is associated with the satisfaction of students’ basic psychological needs and thereby with greater academic buoyancy, which is in turn associated with higher learning engagement. Academic buoyancy thus plays a crucial mediating role in this relationship.

### 3.3 The present study

Academic buoyancy is an established intervening variable in the belonging–engagement relationship, but the share of that association it carries has not been estimated in a rural boarding population. Studies of the same link report academic self-efficacy and academic emotions as intervening variables (Wang et al., 2022, 2023). Because these candidates have not been measured alongside buoyancy within one model, it remains open whether they index a single appraisal-related capacity or operate independently; estimating the indirect effect carried by buoyancy is a first step toward separating them. For students long separated from family support systems, the school environment becomes crucial for their development, making school belonging particularly significant and playing a more critical role in their learning engagement.

The question also carries practical weight. Belonging and buoyancy imply different interventions, the first directed at the school environment and the second at how students appraise setbacks, so the share of the association each accounts for bears directly on where resource-limited schools should place effort. This exploration contributes to understanding the motivational mechanisms of rural boarding students’ engagement while providing empirical evidence for optimizing psychological support strategies in rural education.

### 3.4 Research questions and hypotheses

This study addresses three research questions.

*Research Question 1*: What are the overall levels of school belonging, academic buoyancy and learning engagement, and what are the relationships between these variables?

*H1a*: School belonging, academic buoyancy and learning engagement will be at moderate levels.

*H1b*: The three variables will show significant positive correlations. Positive associations between school belonging and learning engagement, and between academic buoyancy and learning engagement, have been reported across a range of samples (Gillen-O’Neel, 2021; Thomas and Allen, 2021; Yuan et al., 2025), and SDT’s account of interrelated need satisfaction gives a reason to expect them to co-occur in this population (Yuan et al., 2025; Gillen-O’Neel, 2021).

*Research Question 2*: Are there significant differences in the school belonging, academic buoyancy and learning engagement in terms of gender and grade?

Gender and grade were examined as key demographic variables because they are the most fundamental demographic variables when studying students, and previous studies have indicated significant differences in school belonging, academic buoyancy, and learning engagement across these demographics (Chen et al., 2025; Shida, 2024; Meng et al., 2025; Wang et al., 2025).

*H2a*: There will be a significant gender difference in academic buoyancy, with males scoring higher than females, consistent with previous research (Chen and Mok, 2023; Jin, 2020).

*H2b* and *H2c*: No significant gender differences are hypothesized for school belonging or learning engagement, aligning with findings which suggests gender differences in school belonging are small or absent among Chinese primary school students (Tian et al., 2016).

*H2d*: Learning engagement will increase with grade level, given escalating academic pressure and developmental maturity across high school years.

*H2e*: Academic buoyancy will not differ significantly across grades, consistent with its characterization as a relatively stable characteristic (Fu, 2024).

*H2f*: School belonging will be lower in the senior grades than in Grade 1, on the reasoning that sustained academic pressure erodes it (Furrer and Skinner, 2003; Cupito et al., 2015; Wilkinson-Lee et al., 2011).

*Research Question 3*: Does academic buoyancy mediate the relationship between school belonging and learning engagement?

*H3*: School belonging is expected to be positively associated with academic buoyancy, academic buoyancy positively associated with learning engagement, and the indirect association through academic buoyancy significant while the direct association remains significant, that is, a partial rather than a complete indirect pathway.

## 4 Materials and methods

### 4.1 Participants

Ethical approval was obtained from the Academic Committee of Shaanxi Normal University before data collection. Written informed consent was obtained from the parent or legal guardian of every participating student. Consent forms describing the purpose of the study, the content and length of the questionnaire, the voluntary nature of participation, the right to withdraw at any point without giving a reason, the anonymity of responses and the intended use of the data were distributed to guardians through the class teacher before data collection. Only students whose guardians returned a signed consent form were eligible to take part. Students whose guardians declined or did not respond were not surveyed, and no record was made of which students these were. Before the survey was administered, the trained researchers also obtained written assent from each eligible student individually, after providing a verbal explanation of the study and confirming students understood their right to decline or withdraw at any point without consequence.

Sampling proceeded in two stages. At the school level, one rural boarding high school in Yunnan Province was selected purposively rather than at random, on the grounds that it exhibits the features that define the population of interest: full-week boarding, a predominantly rural catchment, and a student body whose families are largely unable to provide day-to-day academic support. Neither stratified nor snowball sampling was used, and the school-level selection is accordingly described here as purposive rather than probability-based. At the class level, cluster sampling was applied within the selected school, with intact classes as the sampling units; classes were surveyed in full rather than sampling individual students within them. Students were told that participation was voluntary, that their teachers would not see their individual responses, that their answers had no bearing on grades or on any school evaluation, and that they could leave any item blank or stop at any point without giving a reason and without consequence. Data collection was conducted during regular school hours. Trained researchers administered the survey in classroom using standardized instructions, emphasizing voluntary participation and data confidentiality.

A total of 670 students completed the questionnaire in the second half of June 2026, following ethical approval on 17 June 2026. After excluding 13 invalid questionnaires due to incomplete responses, patterned answers, or failed lie-detection items, 657 valid responses were retained (effective rate: 98.1%). The final sample comprised 266 males and 391 females, with 179 Grade 1, 217 Grade 2, and 261 Grade 3 students.

### 4.2 Instruments

This study employed a self-report method, utilizing the following three validated Chinese versions of scales adapted to the Chinese educational context.

School Belonging was measured using the Chinese-revised Psychological Sense of School Membership Scale (PSSM-CR) adapted by Pan et al. (2011). This scale contains 18 items, which employs a 5-point Likert scale from 1 “strongly disagree” to 5 “strongly agree,” with higher total scores indicating a stronger sense of school belonging. In this study, the Cronbach’s α coefficient for the scale was 0.890.

Academic Buoyancy was assessed using the scale originally developed by Martin and Marsh (2008) and subsequently translated and adapted for Chinese students by Sun (2009). The unidimensional scale which comprises 4 items employs a 5-point Likert format from 1 “strongly disagree” to 5 “strongly agree,” with higher total scores indicating greater academic buoyancy. In this study, the scale’s Cronbach’s *α* coefficient was 0.727.

Learning Engagement was evaluated using the Utrecht Work Engagement Scale-student (UWES-S) revised by Fang et al. (2008). The scale contains 17 items and uses a 7-point Likert scale, from 1 “never” to 7 “always,” with higher total scores indicating higher levels of learning engagement. In this study, the Cronbach’s *α* coefficient for the scale was 0.957.

### 4.3 Data analysis

Analyses were conducted in SPSS 27.0. Missing values were random and affected fewer than 1% of data points; these were replaced by the item mean. The analysis proceeded in three stages. First, descriptive statistics and Pearson correlations were computed to address H1a and H1b, with 95% confidence intervals for correlations obtained through Fisher’s *z* transformation. Second, independent-samples *t*-tests and one-way ANOVAs were used to test gender and grade differences (H2a to H2f), with Cohen’s *d* and *η*^2^ reported as effect sizes together with 95% confidence intervals for *d*. Post-hoc comparisons among grades used the Games-Howell procedure, which assumes neither equal variances nor equal group sizes and applies the Welch-Satterthwaite correction to the degrees of freedom for each contrast; it was chosen because the grade groups were unequal in size (*n* = 179, 217 and 261) and Levene’s test indicated heterogeneity of variance across grades for school belonging (*F* (2, 654) = 17.679, *p* < 0.001), conditions under which it controls Type I error better than Tukey’s HSD (Games and Howell, 1976). Third, the indirect association was estimated with PROCESS Model 4 using percentile bootstrap confidence intervals based on 5,000 resamples, controlling for gender and grade.

### 4.4 Common method bias assessment

Given that all key variables were measured by student self-report in a single session, we assessed the potential threat of common method bias using Harman’s single-factor test (Podsakoff et al., 2003). An unrotated exploratory factor analysis including all items from the study’s scales yielded a Kaiser-Meyer-Olkin (KMO) measure of 0.828, indicating excellent sampling adequacy, and Bartlett’s test of sphericity was significant (*χ*^2^ = 2132.521, *p* < 0.001). The first unrotated factor explained 49.37% of the total variance, which is below the conventional threshold of 50%. This suggests that while common method variance is present, it is unlikely to be severe enough to account for the majority of covariance among the measures or to substantively distort the observed relationships. As noted in the limitations, this test alone cannot fully rule out common method bias.

## 5 Results

### 5.1 Tests of normality

Normality was assessed for the three main variables using the Shapiro–Wilk test, supplemented by examination of skewness and kurtosis coefficients. The Shapiro–Wilk tests were statistically significant for all variables (*p* < 0.05), indicating rejection of the null hypothesis of normal distribution. However, examination of the distributional characteristics showed that all absolute skewness values were below 2 (ranging from −0.503 to 0.071) and all absolute kurtosis values were below 7 (ranging from −0.600 to 0.807), which fall within acceptable ranges for assuming approximate normality in applied research. Furthermore, with a large sample size well above 500, the Central Limit Theorem ensures the robustness of parametric statistical analyses to such minor deviations from normality.

### 5.2 Descriptive statistics and correlations

All three variables were at moderate levels (see Table 1): school belonging (*M* = 3.020, SD = 0.458), academic buoyancy (*M* = 3.252, SD = 0.802) and learning engagement (*M* = 4.274, SD = 1.165). All pairwise correlations were positive and significant: school belonging with learning engagement (*r* = 0.436, 95% CI [0.372, 0.496]), academic buoyancy with learning engagement (*r* = 0.380, 95% CI [0.313, 0.444]), and school belonging with academic buoyancy (*r* = 0.286, 95% CI [0.214, 0.355]). H1a and H1b were supported.

**Table 1:** Descriptive statistics and correlations of major variables (*N* = 657).

Variables	*M*	Max	Min	SD	1	2	3
1. School belonging	3.020	5	1	0.458	—		
2. Learning engagement	4.274	7	1	1.165	0.436**	—	
3. Academic buoyancy	3.252	5	1	0.802	0.286**	0.380**	—

*M*, mean; SD, standard deviation; **p < 0.01. 95% confidence intervals for the correlations are reported in the text.

### 5.3 Gender and grade differences

To test hypothesis H2, independent samples t-tests and one-way ANOVA were conducted to examine gender and grade differences. The t-test results (see Table 2) indicated that male students scored significantly higher than female students in academic buoyancy (*t* (655) = 4.523, *p* < 0.001, Cohen’s *d* = 0.359, 95% CI [0.202, 0.516]), supporting H2a. No significant gender differences were found for school belonging (*t* (655) = 0.492, *p* = 0.623, *d* = 0.039, 95% CI [−0.117, 0.195]) or learning engagement (*t* (655) = −0.814, *p* = 0.416, *d* = −0.065, 95% CI [−0.221, 0.091]), supporting H2b and H2c.

**Table 2:** Gender and grade differences in the major variables (*N* = 657).

Variable	Group	*M* ± SD	*t*/*F*	*p*	Effect size [95% CI]
School belonging	Boys (*n* = 266)	3.030 ± 0.486	0.492	0.623	*d* = 0.039 [−0.117, 0.195]
	Girls (*n* = 391)	3.012 ± 0.439			
	Grade 1 (*n* = 179)	3.117 ± 0.563	5.687	0.004	*η*^2^ = 0.017
	Grade 2 (*n* = 217)	2.979 ± 0.360			
	Grade 3 (*n* = 261)	2.987 ± 0.443			
Academic buoyancy	Boys (*n* = 266)	3.421 ± 0.790	4.523	<0.001	*d* = 0.359 [0.202, 0.516]
	Girls (*n* = 391)	3.137 ± 0.791			
	Grade 1 (*n* = 179)	3.218 ± 0.816	2.312	0.100	*η*^2^ = 0.007
	Grade 2 (*n* = 217)	3.347 ± 0.782			
	Grade 3 (*n* = 261)	3.196 ± 0.806			
Learning engagement	Boys (*n* = 266)	4.230 ± 1.160	−0.814	0.416	*d* = −0.065 [−0.221, 0.091]
	Girls (*n* = 391)	4.305 ± 1.168			
	Grade 1 (*n* = 179)	3.886 ± 1.228	15.158	<0.001	*η*^2^ = 0.044
	Grade 2 (*n* = 217)	4.343 ± 1.090			
	Grade 3 (*n* = 261)	4.484 ± 1.118			

*M*, mean; SD, standard deviation. For gender comparisons the test statistic is *t* (655); for grade comparisons *F* (2, 654). Cohen’s *d* with 95% CI for gender comparisons; *η*^2^ for grade comparisons. Post-hoc comparisons used the Games-Howell procedure.

Grade differences in school belonging were significant (*F* (2, 654) = 5.687, *p* = 0.004, *η*^2^ = 0.017). Games-Howell comparisons showed that Grade 1 students (*M* = 3.117, SD = 0.563) scored higher than both Grade 2 students (*M* = 2.979, SD = 0.360; *p* = 0.013, *d* = 0.30) and Grade 3 students (*M* = 2.987, SD = 0.443; *p* = 0.026, *d* = 0.26), H2f, which predicted lower school belonging in the senior grades than in Grade 1, was supported. The difference takes the form of a drop after Grade 1 followed by a plateau rather than a continuing decline, since Grade 2 and Grade 3 did not differ significantly.

Grade differences in learning engagement were also significant (*F* (2, 654) = 15.158, *p* < 0.001, *η*^2^ = 0.044). Games-Howell comparisons showed that Grade 1 students (*M* = 3.886, SD = 1.228) scored lower than both Grade 2 students (*M* = 4.343, SD = 1.090, *p* < 0.001) and Grade 3 students (*M* = 4.484, SD = 1.118, *p* < 0.001), while Grade 2 and Grade 3 did not differ significantly. H2d predicted an increase across grade levels and is therefore partially supported: engagement is higher after Grade 1, but it does not continue to rise thereafter.

Grade differences in academic buoyancy were not significant (*F* (2, 654) = 2.312, *p* = 0.100, *η*^2^ = 0.007), and no pairwise comparison reached significance. H2e was supported.

Because gender was associated with academic buoyancy and grade with school belonging and learning engagement, both were entered as covariates in the analysis that follows.

### 5.4 The mediating role of academic buoyancy

We used PROCESS Macro Model 4 to examine the mediating role of academic buoyancy between school belonging and learning engagement, controlling for gender and grade.

The regression results (see Table 3) revealed that school belonging significantly predicted academic buoyancy (*B* = 0.491, SE = 0.065, beta = 0.281, *t* = 7.527, *p* < 0.001) and learning engagement (*B* = 0.967, SE = 0.085, beta = 0.382, *t* = 11.313, *p* < 0.001). Academic buoyancy also significantly predicted learning engagement (*B* = 0.429, SE = 0.049, beta = 0.295, *t* = 8.706, *p* < 0.001). The model for academic buoyancy accounted for 11.0% of the variance and the model for learning engagement for 33.4%.

**Table 3:** Regression models for academic buoyancy and learning engagement (*N* = 657).

Outcome variable	Predictor	*R*	*R* ^2^	*F*	*B* (SE)	*t*	*p*	Beta
Academic buoyancy		0.332	0.110	20.22***				
	School belonging				0.491 (0.065)	7.527	<0.001	0.281
	Gender				−0.275 (0.061)	−4.549	<0.001	−0.169
	Grade				−0.003 (0.037)	−0.074	0.941	−0.003
Learning engagement		0.578	0.334	65.16***				
	School belonging				0.967 (0.085)	11.313	<0.001	0.382
	Academic buoyancy				0.429 (0.049)	8.706	<0.001	0.295
	Gender				0.261 (0.077)	3.374	0.001	0.110
	Grade				0.368 (0.046)	7.915	<0.001	0.256

*B* = unstandardized regression coefficient, with the standard error in parentheses; beta = standardized regression coefficient. Gender was coded 1 = male, 2 = female; grade was entered as 1, 2, 3. ***p < 0.001.

Figure 1 shows the standardized coefficients for this model. Percentile bootstrap confidence intervals based on 5,000 resamples are reported in Table 4. The total effect of school belonging on learning engagement was *B* = 1.178, SE = 0.087, 95% CI [1.008, 1.348], standardized effect 0.464. The indirect effect through academic buoyancy was *B* = 0.211, SE = 0.038, 95% CI [0.140, 0.291], which excludes zero; the completely standardized indirect effect was 0.083. The direct effect remained significant, *B* = 0.967, SE = 0.086, 95% CI [0.800, 1.135], standardized effect 0.381. The indirect effect accounted for 17.87% of the total effect. H3, which specified a significant indirect effect alongside a significant direct effect, was supported.

**Figure 1:**
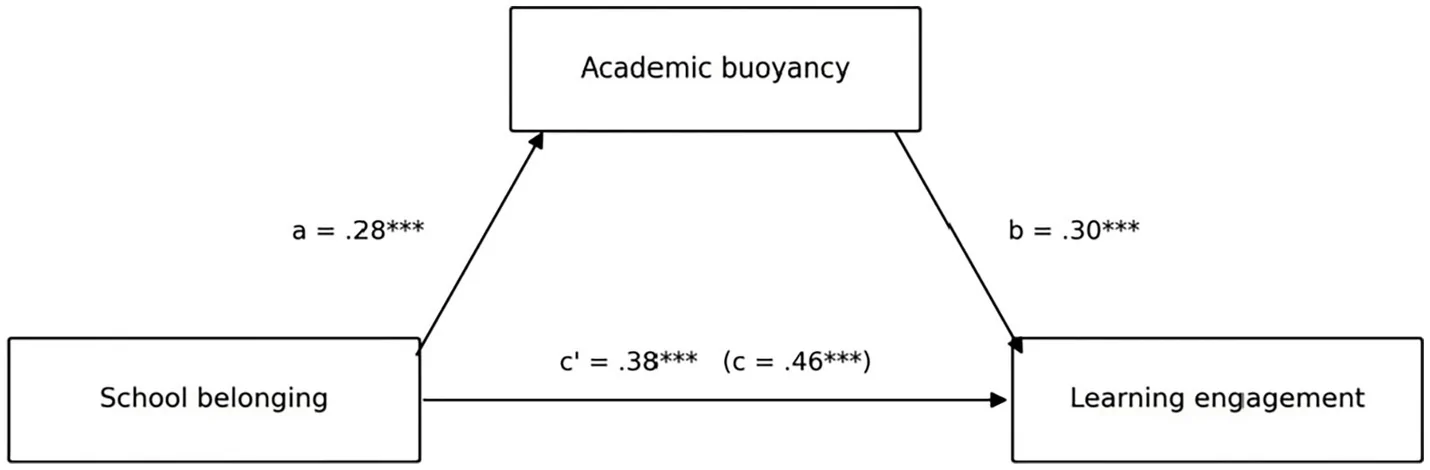
Path analysis results. Standardized path coefficients for the model with academic buoyancy as the intervening variable (*N* = 657), controlling for gender and grade. All coefficients are standardized (beta). *c* = total effect of school belonging on learning engagement; *c*′ = direct effect with academic buoyancy in the model. ***p < 0.001.

**Table 4:** Decomposition of the total, direct and indirect effects of school belonging on learning engagement.

Effect	*B* (unstd.)	SE	Boot LLCI	Boot ULCI	% of total	Standardized effect
Total effect	1.1779	0.0866	1.0078	1.3479	100%	0.4636
Direct effect	0.9674	0.0855	0.7995	1.1353	82.13%	0.3807
Indirect effect	0.2105	0.0381	0.1402	0.2906	17.87%	0.0828

*B* = unstandardized coefficient; Boot LLCI and Boot ULCI are the bounds of the bias-corrected bootstrap 95% confidence interval based on 5,000 resamples. The final column reports the completely standardized effect. Gender and grade were entered as covariates.

## 6 Discussion

### 6.1 Overall levels and correlations

This study found that the school belonging, academic buoyancy, and learning engagement of high school students in rural boarding schools were all at a medium level. Further correlation analysis indicated there were significant pairwise positive correlations among school belonging, academic buoyancy and learning engagement. The correlations point in the same direction and are of comparable magnitude to those reported in Chinese vocational-college samples (Wang et al., 2022; Wang et al., 2023) and in a daily-diary study of college students (Gillen-O’Neel, 2021), and the buoyancy-engagement association is consistent with results from Australian, American and Finnish samples (Martin et al., 2010; Thomas and Allen, 2021; Ursin et al., 2021).

### 6.2 Gender and grade differences

Male students reported higher academic buoyancy than female students, with no gender difference in belonging or engagement. The direction replicates findings from a Chinese high school sample (Jin, 2020) and a Chinese adolescent sample (Chen and Mok, 2023). The absence of a gender difference in belonging is consistent with what has been reported among Chinese students, although in a primary school sample (Tian et al., 2016); direct evidence on gender differences in learning engagement among Chinese boarding high school students remains limited. Because the same direction appears in day-school samples, the difference is unlikely to be produced by boarding arrangements as such.

The pattern is informative precisely because it is selective. If a general disposition such as global resilience were driving the results, one would expect gender differences to appear across the outcomes; that they appear only for buoyancy is more consistent with buoyancy being a domain-specific appraisal of one’s capacity to handle academic setbacks, in the sense intended by Martin and Marsh (2008), than with a broad trait.

Regarding grade differences, significant differences were found in school belonging, with Grade 1 students scoring highest and Grades 2 and 3, which did not differ from each other, scoring lower. Furrer and Skinner (2003) reported that children’s sense of relatedness to their teachers dropped significantly following the transition to middle school in Grade 6, and lower school belonging at higher grade levels has also been reported (Tian et al., 2016; Cupito et al., 2015; Wilkinson-Lee et al., 2011). That is to say, the higher the grade, the lower the school belonging. Consistent with this trend, Grade 1 students in our study reported higher belonging than their counterparts in Grades 2 and 3. However, the decline did not continue across the senior grades, so belonging in this setting does not fall linearly through the boarding high school years. These are differences between grade cohorts measured at a single time rather than change within students. The pattern can nonetheless be understood through shifts in the fulfillment of basic psychological needs across grades. The sense of belonging in Grade 1 stems primarily from the initial satisfaction of the need for relatedness. Entering a new collective provides students with a clear social identity, forming the basis of their belonging.

The lower belonging of the senior grades likely stems from frustrated competence needs undermining relational security. Increased academic demands persistently challenge students’ sense of mastery. Within the boarding context, characterized by prolonged separation from family support, students have fewer emotional resources to buffer these setbacks. The recurrent feeling of “I cannot handle this” not only damages their perceived competence but also erodes their foundational sense of belonging. In this closed environment where achievement is centrally valued, ongoing academic pressure thus weakens students’ identification with and perceived value within the school community.

That belonging holds steady across the senior grades rather than falling further suggests a shift in its source. Under the overriding pressure of the college entrance exam, peer connections may transform from generalized emotional identification into a pragmatic symbiotic relationship oriented toward a shared goal. This form of bonding, based on collective striving, resonates with the emphasis on group objectives within collectivist culture, thereby sustaining a particular form of belonging even under external high pressure.

Regarding learning engagement, significant grade differences were observed, with Grade 1 lowest and the senior grades higher. The rise in engagement, contrasted with belonging moving in the opposite direction, suggests that the primary driver in senior years may be external pressure, specifically from the college entrance exam, rather than belonging itself. While belonging offers a foundational motivational base, the overwhelming importance of this exam acts as a powerful external driver. According to SDT, motivation exists along a continuum from controlled to autonomous regulation (Ryan and Deci, 2017). This framework helps interpret the motivational landscape in this study: engagement in higher grades likely reflects a mixture along this continuum. In Grade 2, engagement may be sustained more by introjected or external regulation, such as emerging pressure and fear of failure, compensating for lowered belonging-driven volition. By Grade 3, engagement appears predominantly externally regulated by the exam’s salience. Thus, while both belonging and exam pressure co-exist in the final year, they energize engagement through qualitatively different motivational processes: belonging supports more internalized forms of regulation, whereas the exam exerts strong external control. Consequently, the high engagement observed in Grade 3 does not equate to high autonomous motivation. This also bears on the mediation result, since two variables that move in opposite directions across grades cannot stand in a simple driving relationship.

The absence of significant grade differences in academic buoyancy aligns with Jin’s (2020) findings and supports the characterization of academic buoyancy as a relatively stable personal resource (Fu, 2024). The intervening variable in the model is thus the one variable that does not vary with grade, while the two variables it connects both do.

### 6.3 The mediating role of academic buoyancy

The mediation analysis confirms that academic buoyancy plays a significant partial mediating role between school belonging and learning engagement, with the direct association remaining significant. Because the three variables were measured at one time, the result describes a statistical indirect association rather than a causal sequence. Read in SDT terms, it is consistent with a pathway in which school belonging, by supporting the satisfaction of basic psychological needs, is associated with a greater capacity to cope with academic setbacks, which is in turn associated with sustained learning engagement.

Partial rather than complete mediation has also been reported for the same link with different intervening variables, namely academic self-efficacy and academic emotions in Chinese vocational-college samples (Wang et al., 2022; Wang et al., 2023), so three different intervening variables measured in different populations each carry part of the association and none carries all of it. The more economical account is not that each names a separate mechanism but that they are overlapping indicators of a common appraisal-related capacity that belonging supports and that engagement draws on, an overlap consistent with network-analytic work on buoyancy and coping (Putwain et al., 2024). Control appraisals (Collie et al., 2017) and teacher support together with emotional reactivity (Pitzer and Skinner, 2017) are further candidates identified in samples where belonging was also measured, and separating them from academic buoyancy would require these variables to be measured within one model.

The identified pathway from emotional connection, through the capacity to handle academic setbacks, to behavioral engagement underscores that school belonging is associated with engagement both directly and through students’ capacity to navigate academic challenges. Taken with the grade and gender results, the picture is coherent rather than a set of separate observations: academic buoyancy differs by gender where belonging and engagement do not, and it is flat across grades where the other two diverge, so it is not interchangeable with either and cannot be the route through which grade-related pressure reaches engagement. The association therefore runs partly through a stable, domain-specific capacity and partly through channels that vary with the pressure students face at each stage, which is why fostering both a sense of belonging and the capacity to absorb setbacks addresses different portions of the same association, particularly in resource-limited rural boarding contexts.

### 6.4 Contributions and limitations

This study makes two principal contributions. Theoretically, it examines academic buoyancy as an intervening variable in the statistical association between school belonging and learning engagement and quantifies the share of the association it accounts for, adding to the limited body of work on the mechanisms linking these two constructs in Chinese secondary samples. Regarding the research subjects, by focusing on high school students in resource-limited rural boarding schools, this study addresses a significant gap in the literature concerning this specific population. The conclusions offer valuable insights and targeted references for promoting educational equity in similar contexts.

However, some limitations in this study should be acknowledged. First, the cross-sectional design precludes causal inference. Although the hypothesized mediation model is theory-grounded and statistically supported, longitudinal or experimental designs are required to establish temporal precedence among school belonging, academic buoyancy, and learning engagement. Second, the reliance on self-reported data collected at a single time point raises concerns regarding common method variance. Harman’s single-factor test indicated that common method variance was not severe; however, this test alone is insufficient to rule out the concern, and future research would benefit from employing multi-method assessments—such as teacher ratings and behavioral observations—and multi-wave data collection to triangulate findings. Third, all participants were recruited through cluster sampling of intact classes from a single rural boarding school in Yunnan Province; replication across diverse geographical and institutional contexts through longitudinal and intervention-based designs is necessary to assess the broader applicability and causal validity of the model. Fourth, consistent with its role as an interpretive framework, Self-Determination Theory’s three basic psychological needs—autonomy, competence, and relatedness—were not directly measured in this study. The proposed pathway from relatedness satisfaction through buoyancy to engagement therefore remains theoretically grounded but unconfirmed; future work should include direct measures of all three needs within a single model to test the SDT account more rigorously.

## 7 Conclusion and implications

### 7.1 Conclusion

Among 657 students at a rural Chinese boarding high school, school belonging, academic buoyancy and learning engagement were at moderate levels and positively correlated, and academic buoyancy accounted for a significant but modest share of the association between school belonging and learning engagement, with the direct association remaining significant. Male students reported higher academic buoyancy than female students, with no gender differences in the other two variables. School belonging was highest in Grade 1 and lower in the senior grades, while learning engagement showed the opposite pattern; academic buoyancy did not differ across grades. The grade comparisons are between cohorts rather than a developmental trajectory. These findings describe statistical associations consistent with the theoretical model rather than a demonstrated causal sequence, and underscore the need for context-sensitive interpretations of motivation in high-pressure, collectivist educational settings like rural Chinese boarding schools.

### 7.2 Implications

The findings of this study provide clear dual-track intervention ideas and practical paths for enhancing the learning engagement of rural boarding high school students. Educational practice should focus on both emotional connection and academic buoyancy cultivation. On the one hand, it is necessary to enhance students’ sense of school belonging by establishing a supportive school atmosphere, such as developmental mentorship programs and strengthening teacher-student and peer interactions. On the other hand, it is also necessary to directly strengthen students’ academic buoyancy through psychological skills training, such as attribution training and coping strategies for setbacks.

The grade findings suggest where to concentrate effort, on the understanding that these are between-cohort differences. The transition out of Grade 1 is the point at which belonging drops and engagement rises, so it is the natural target for intervention: the practical question is whether the sense of membership that Grade 1 students bring with them can be maintained as academic demands increase, rather than being traded for externally driven engagement. Because Grades 2 and 3 did not differ on either variable, there is no evidence here for treating the final year as a distinct case in terms of belonging or engagement levels. For Grade 1 students, group activities that build on the initial sense of collective membership can help sustain belonging as academic pressure increases. For the senior grades, advisory programs and peer support structures address the lower belonging without relying on the examination as the sole driver of engagement.

Finally, it is essential to integrate resources from both families and schools to compensate for gaps in students’ emotional support systems. Establishing a learning promotion framework grounded in emotional support, with psychological resilience as the bridge, can effectively enhance students’ learning engagement and foster positive development, even within resource-constrained environments.

## Data Availability

The original contributions presented in the study are included in the article/supplementary material, further inquiries can be directed to the corresponding author.
